# Preterm Delivery; Who Is at Risk?

**DOI:** 10.3390/jcm10112279

**Published:** 2021-05-24

**Authors:** Dvora Kluwgant, Tamar Wainstock, Eyal Sheiner, Gali Pariente

**Affiliations:** 1 Soroka University Medical Center, Department of Obstetrics and Gynecology, Faculty of Health Sciences, Ben-Gurion University of the Negev, Beer-Sheva 8400711, Israel; feinblum@post.bgu.ac.il (D.K.); galipa@bgu.ac.il (G.P.); 2 School of Public Health, Ben-Gurion University of the Negev, Beer-Sheva 8400711, Israel; wainstoc@bgu.ac.il

**Keywords:** preterm birth, extreme preterm birth, placental abruption, prematurity

## Abstract

Preterm birth (PTB) is the leading cause of perinatal morbidity and mortality. Adverse effects of preterm birth have a direct correlation with the degree of prematurity, in which infants who are born extremely preterm (24–28 weeks gestation) have the worst outcomes. We sought to determine prominent risk factors for extreme PTB and whether these factors varied between various sub-populations with known risk factors such as previous PTB and multiple gestations. A population-based retrospective cohort study was conducted. Risk factors were examined in cases of extreme PTB in the general population, as well as various sub-groups: singleton and multiple gestations, women with a previous PTB, and women with indicated or induced PTB. A total of 334,415 deliveries were included, of which 1155 (0.35%) were in the extreme PTB group. Placenta previa (OR = 5.8, 95%CI 4.14–8.34, *p* < 0.001), multiple gestations (OR = 7.7, 95% CI 6.58–9.04, *p* < 0.001), and placental abruption (OR = 20.6, 95%CI 17.00–24.96, *p* < 0.001) were the strongest risk factors for extreme PTB. In sub-populations (multiple gestations, women with previous PTB and indicated PTBs), risk factors included placental abruption and previa, lack of prenatal care, and recurrent pregnancy loss. Singleton extreme PTB risk factors included nulliparity, lack of prenatal care, and placental abruption. Placental abruption was the strongest risk factor for extreme preterm birth in all groups, and risk factors did not differ significantly between sub-populations.

## 1. Introduction

Preterm delivery, defined as delivery prior to 37 weeks of gestation, is a leading cause of perinatal morbidity and mortality worldwide, with an incidence of 5–13% depending on location [1]. Since the prevalence of preterm delivery is so high, it is thought to put more financial, medical, and emotional stress on affected communities than any other perinatal issue [2]. Additionally, prematurity has both short and long-standing consequences for affected infants and can leave these individuals with lifelong disabilities, even after the available interventions are attempted [3,4,5]. Morbidity and mortality are higher among those defined as “very” preterm (<32 weeks) and “extremely” preterm (<28 weeks), but prognosis has improved in recent years with better care, even among those born at 22–23 weeks [6,7,8,9]. However, it should be noted that this varies between countries.

Many factors can predispose to the development of preterm delivery, but it is useful to categorize preterm birth into three general etiologic groups: spontaneous labor with intact membranes, preterm premature rupture of membranes (PPROM) leading to preterm delivery, and labor induction due to maternal or fetal factors [4,10]. These categories each have their own common risk factors; for example, risk factors for PPROM-induced delivery include intrauterine infection [11], tobacco use [12], abruption [13], multiple gestations [14], previous PPROM [15], and cervical factors [16,17], among others. However, these risk factors are not exclusive to each etiologic group, and some can be risk factors for multiple groups.

Other risk factors include previous C section [18], low pre-pregnancy BMI [19], and hypertensive disorders of pregnancy [20]. Women who have had one previous spontaneous preterm delivery have an increased risk of subsequent preterm delivery in their next pregnancy, with an absolute risk of about 30% of another preterm delivery [21,22]. Primigravid women and those carrying male fetuses also have a higher association with preterm delivery [2]. Even seemingly insignificant factors such as ambient air temperature can have an impact on rates of preterm delivery [23].

While various risk factors for preterm delivery are well recognized, it is still unclear whether the cause of preterm delivery is multifactorial, or whether each risk factor leads to a different pathophysiologic cause of preterm delivery [1]. In this study, we attempt to evaluate the impact of different known risk factors on the occurrence of extremely preterm birth (<28 weeks) while controlling for confounders. We also examine whether different risk factors for preterm delivery were more important in various subgroups, such as induced versus spontaneous preterm birth and multiple versus singleton gestations.

## 2. Materials and Methods

### 2.1. Population and Study Design

This retrospective cohort study was performed using data from the birthing center at Soroka University Medical Center (SUMC). SUMC is the largest tertiary hospital in the Negev region of Israel and serves the entire population of this area. Data were collected using the computerized perinatal database. Information from the perinatal database is first documented directly following delivery by an attending physician. Subsequently, medical secretaries routinely review the information before it is entered into the database. After evaluating prenatal care records together with the routine hospital documents, coding is performed. These measures ensure maximal completeness and accurateness of the databases. The databases include demographic information and International Classification of Diseases, 9th revision codes (ICD-9) for all diagnoses. The institutional review board, in accordance with the Helsinki declaration, approved the study (0358-19-SOR). All deliveries between the years 1991 and 2018 were included. Cases of fetal malformations or chromosomal abnormalities of the fetus were excluded from the study. 

Four groups were examined based on gestational age, as put forth by the WHO: extreme preterm (24 + 0–27 + 6 weeks), very preterm (28 + 1–31 + 6 weeks), and moderate to late preterm (32 + 0–36 + 6 weeks), with a reference group of term births (>37 + 0 weeks) [24]. We examined the following obstetric risk factors and evaluated their impact on the occurrence of preterm birth in different gestational ages while controlling for confounders: maternal age, ethnicity, nulliparity, previous cesarean delivery, recurrent pregnancy loss, diabetes mellitus, hypertension, use of in vitro fertilization or ovarian induction, lack of prenatal care, gestational diabetes mellitus, preeclampsia, placenta previa, placental abruption, as well as delivery characteristics such as cesarean delivery, assisted delivery, maternal need for blood transfusions after delivery, post-partum hemorrhage, and fetal characteristics such as fetal gender, small for gestational age fetus, 5-min APGAR score <7, umbilical cord pH <7, intrapartum death, and number of fetuses. Placental abruption was clinically defined as the premature detachment of an implanted placenta from the uterine wall before the delivery of the fetus. The diagnosis was made by the attending staff during the delivery [25,26]. In some of the cases, the diagnosis was confirmed by pathological examination. Nevertheless, as abruption is considered a clinical diagnosis, only some cases of acute abruptions demonstrated histologic confirmation. Maternal exposure, with or without placenta abruption, as well as all other clinical characteristics were identified using ICD-9 codes, with ICD-9 code 641.2 for placental abruption. Placenta previa occurs when the placenta attaches inside the uterus but in an abnormal position near or over the cervical opening [27]. As the diagnosis of placenta previa may change later in pregnancy, we defined it here as placenta previa diagnosed on ultrasound before delivery during routine ultrasounds, which in Israel are performed at 14, 22, and 30 weeks, and also every time a woman presents for prenatal care.

We further examined whether different risk factors and outcomes for preterm delivery were more important in various subgroups; induced vs. spontaneous preterm birth, those with vs. without previous PTB, and multiples vs. singletons. The vast majority of multiples born at our center were twins (65.5%), with the remainder being triplets (6.6%) or quadruplets (0.7%). Therefore, we did not differentiate between twins and higher order multiples, since the rates of higher order multiples were exceedingly low (twins = 3.4%, triplets = 0.1%).

### 2.2. Statistical Analysis

Statistical analysis was performed using SPSS (SPSS, Chicago, IL, USA). We used the Chi-square test to calculate the statistical significance based on differences between qualitative variables and the *t* for continuous variables. Few multivariable logistic regression models were created in order to examine independent risk factors for preterm delivery according to gestational age and among different sub-groups, while controlling for confounders. Odds ratios and their 95% confidence intervals were calculated, and *p*-values less than 0.05 were considered statistically significant.

## 3. Results

A total of 334,415 births were included in our study, including extreme PTB (*n* = 1155), very PTB (*n* = 2490), moderate-late PTB (*n* = 25,344), and term births (*n* = 304,732). Characteristics of the overall population are summarized in Table 1. There was a significantly higher rate of PTB in Bedouin women, especially in the very PTB group (57.1%, *p* < 0.001). The rate of nulliparity was significantly higher in the extreme PTB group (37.3%, *p* < 0.001). Rates of recurrent pregnancy loss, lack of prenatal care, placenta previa and abruption, need for maternal blood transfusion, postpartum hemorrhage, small for gestational age neonates, 5-min APGAR score <7, umbilical cord pH <7, and intrapartum death were also all significantly higher in the extreme PTB group than in all others. Those in the very preterm group had the highest rates of hypertension, use of in vitro fertilization and ovulation induction, preeclampsia, delivery by cesarean delivery, multiple gestations, and male fetal gender. Rates of previous cesarean delivery were 25.7%, 27.9%, 25.9%, and 15.3% for extreme PTB, very PTB, moderate–late PTB, and term deliveries, respectively *p* < 0.001. Rates of previous PTB were 27.5%, 30.1%, 28.8%, and 11.1% for extreme PTB, very PTB, moderate–late PTB, and term deliveries, respectively *p* < 0.001. Rates of small for gestational age infants were 14.2%, 6.2%, 4.3%, and 4.7% among extreme PTB, very PTB, moderate PTB, and term deliveries, respectively, *p* < 0.001. Rates of large for gestational age infants were 0.1%, 0.2%, 0.4%, and 4.8% among extreme PTB, very PTB, moderate PTB, and term deliveries, respectively, *p* < 0.001. Finally, those in the moderate–late PTB group had the highest rates of diabetes mellitus and gestational diabetes mellitus, while the term births had the highest percentage of Jewish mothers and female neonates.

Logistic regression (Table 2) showed placental abruption to be the most significant independent risk factor in the extreme PTB group (OR = 13.579, CI = 8.757–21.057, *p* < 0.001). Other factors that were independent risk factors in this gestational age group were lack of prenatal care, nulliparity, placenta previa, recurrent pregnancy loss, induction of labor, and multiple gestation. In the other PTB groups (Table 3), abruption was also the most significant risk factor (OR = 22.799, CI = 18.422–28.216, *p* < 0.001). Interestingly, having a history of diabetes mellitus (OR = 0.362, CI = 0.238–0.552, *p* < 0.001) decreased the probability of PTB in this gestational age group. Adding the child’s year of birth to the logistic regression model did not significantly affect the results of the model. The only variable that lost its significance as risk factor for extreme PTB was preeclampsia.

In the extreme PTB group (Table 3), those who had a history of previous PTB had the highest rates of Bedouin ethnicity, placenta previa, need for maternal blood transfusion, postpartum hemorrhage, female fetal gender, small for gestational age neonates, and 5-min APGAR <7. The other PTB groups in those with a history of PTB, the highest rates of previous cesarean delivery, diabetes mellitus, hypertension, use of in vitro fertilization and ovulation induction, gestational diabetes mellitus, preeclampsia, cesarean delivery, male neonatal gender, and intrapartum death were seen. Notably, term babies born to women both with and without a history of PTB had the highest rates of assisted delivery. Similar to those in the general population, placental abruption was the highest risk factor for PTB (OR = 13.579, CI = 8.757–21.057, *p* = 0.001) in this population according to multivariate analysis (Table 4). Placental abruption was found to be more common among pregnancies with preeclampsia compared to pregnancies without preeclampsia (1.7% vs. 0.5%, OR 3.3, 95% CI 2.93–3.86, *p* < 0.001). A positive non-parametric correlation was demonstrated between the two variables, even though the correlation was very weak.

In singleton deliveries, the rates of nulliparity (Table 5), history of recurrent pregnancy loss, use of in vitro fertilization, lack of prenatal care, placenta previa, abruption, need for maternal blood transfusion, postpartum hemorrhage, small for gestational age neonate, 5-min APGAR <7, and intrapartum death were all highest in the extreme PTB group. For the other PTB groups, there were higher rates of Bedouin ethnicity, previous PTB and cesarean delivery, diabetes mellitus, hypertension, use of ovulation induction, gestational diabetes mellitus, preeclampsia, cesarean delivery, and assisted delivery.

According to logistic regression (Table 6), the greatest risk factor for PTB in this group was placental abruption (OR = 24.619, CI = 20.063–30.210, *p* < 0.001). 

In multiple gestations (Table 7), rates of nulliparity, placenta previa, placental abruption, need for maternal blood transfusion, small for gestational age neonate, 5-min APGAR < 7, and intrapartum death were highest in the extreme PTB group. The other PTB groups had the highest rates of Bedouin ethnicity, previous PTB and cesarean delivery, diabetes mellitus, hypertension, use of in vitro fertilization or ovulation induction, lack of prenatal care, gestational diabetes mellitus, preeclampsia, cesarean delivery, male neonatal gender, and umbilical cord pH > 7. Term babies in both single and multiple gestations had the highest rates of assisted deliveries. In the logistic regression (Table 8), placental abruption was the most significant risk factor for extreme PTB in multiple gestations (OR = 7.467, CI = 4.398–12.677, *p* < 0.001). Those with diabetes mellitus and preeclampsia had a negative risk for extreme PTB (OR = 0.280, CI = 0.131–0.598, *p* = 0.001; OR = −0.229, CI = 1.779 = 12.819, *p* < 0.001, respectively). We did not differentiate between twins and higher-order multiples, since the rates of higher-order multiples were exceedingly low (twins = 3.4%, triplets = 0.1%). 

In those with induced PTB (Table 9), higher rates of Jewish ethnicity, recurrent pregnancy loss, use of in vitro fertilization, placenta previa, need for maternal blood transfusion, postpartum hemorrhage, small for gestational age neonate, 5-min APGAR < 7, and intrapartum death were seen in the extreme PTB group. In the other induced PTB groups, higher rates of previous PTB and cesarean delivery, diabetes mellitus, hypertension, use of ovulation induction, lack of prenatal care, preeclampsia, cesarean delivery, maternal blood transfusion, and multiple gestations were seen. In the logistic regression (Table 10), the most important risk factor for induced extreme PTB was abruption (OR = 14.175, CI = 8.654–23.218, *p* < 0.001). Diabetes mellitus had a negative predictive value for induced extreme PTD (OR = 0.262, CI = 0.123–0.556, *p* < 0.001). Adding child’s year of birth to the logistic regression models of other sub-populations (previous PTB, singleton pregnancies, multiple gestations and indicated PTBs) did not affect significantly the results of the models.

## 4. Discussion

The results of our study add to the growing body of information on this topic and provide data specific to the population under study, leaving room for further investigation. In this study, the most notable outcome we found was that placental abruption (defined here as clinically diagnosed placental abruption) was the risk factor with the highest significance in all of the populations and sub-populations (e.g., early PTB multiples, induced early PTB, etc.) that we looked at. As placental abruption is a clinical diagnosis, its association with induction of labor may be due to the clinical decision to induce labor following a suspected abruption or may be related to abruption caused by the induction itself. Another interesting finding of our study is that in our population, having diabetes mellitus had an inverse relationship with risk of early PTB. Preeclampsia also showed a weak, negatively predictive effect on extreme PTB, but this was non-statistically significant. Rates of assisted delivery (which at our facility entails use of vacuum extraction) were lower than in other settings as well; these rates are indeed low, since in our medical center, vacuum is hardly performed in preterm deliveries [28].

As is well known and widely noted in other studies, placental abruption is often associated with preterm delivery [10,13]. This risk factor is very significant, with the risk being estimated as between 1.2 and 31.7, and incidence being between 40 and 60% [13]. Placental abruption has been shown by other studies to be nine times more likely to occur in preterm gestational ages than is in term gestational ages (2.8% versus 0.3%, respectively) [29]. Still, other studies have shown that placental abruption implicates itself in 5.8% of births occurring before 35 weeks of gestation, with another finding that 50% of women with PTB had “clinical or histological abruption, chorioamnionitis, or both” [30,31].

Placental abruption is defined as a premature detachment of the placenta from the uterine wall, which occurs after 20 weeks of gestation but before birth. Typical presenting will include vaginal bleeding, abdominal pain, contractions, and abnormal fetal heart rate tracings. Placental abruption leads to uteroplacental under perfusion, hypoxia, and placental ischemia. Thus, abruption can cause a spontaneous PTB, but it may also be an indication for an induced PTB in order to save the life of the mother or her fetus. The mechanism by which placental abruption causes spontaneous PTB is believed to be due to blood irritating the uterine lining and stimulating contractions, which may subsequently lead to PTB [13].

There are a few reasons why placental abruption was found to be the most significant risk factor for PTB in our study. Firstly, abruption was found to have the highest incidence during weeks 24–26 [32], which is a timeframe that falls into the early PTB gestational age group, explaining the high odds ratio seen with abruption in this group. Additionally, over 50% of abruption occurs before term, meaning that if abruption occurs and leads to a natural or induced delivery, it is highly likely that the neonate will be premature. One area our study did not explore was whether the rates of women with risk factors (such as smoking, use of cocaine, etc.) for abruption had a higher rate of PTB caused by abruption, this is an area that is ripe for exploration, as it may help explain our results.

Many studies show that gestational diabetes mellitus and pregestational diabetes mellitus can lead to an increased risk of preterm birth. This being said, there is no consensus as to whether diabetes mellitus is an independent risk factor for spontaneous PTB. Some studies have demonstrated an increased risk of PTB in both pregestational and gestational diabetes [33], while others indicated that this risk was associated only with insulin-treated diabetes mellitus [34]. One study showed obesity to have protective effects against preterm birth [35], which could help explain the results seen in our population, as women with diabetes mellitus are more likely to be obese. 

The uncertainty of the effect of gestational diabetes mellitus in our study may be related to the fact that instead of categorizing diabetes mellitus as gestational versus pregestational, we used a composite outcome that included all cases of diabetes mellitus in pregnancy. Since the mechanism of placental pathology is likely different in these two entities, more clear results may be obtained by studying these as separate pathologies and looking at the effect of each on the risk of PTB. 

The strengths of this study lie in the fact that it was performed using a very large perinatal database with many years of delivery information about mothers and their neonates. This gave us access to a large population from which we could aggregate data and determine the results. Another strength of this study was that we used sub-group analysis, which allowed us to examine each risk factor on its own while controlling for confounders. The most significant limitation to this study is that it was done retrospectively, and therefore, the results do not indicate causation but rather correlation between the risk factors and outcomes. Another weakness lies in the fact that we did not have data to perform distinct analyses on the risks of extreme PTB in monochorionic versus dichorionic twins and rather regarded them as one entity (‘multiple gestations’). Additionally, the rate of extreme PBT in our population was significantly lower than that of other populations. It should be noted that some populations report lower rates of extreme PTB; this may be due to differences among populations [36]. Since our study focused on gestational age, we did not perform any analyses based on birthweight centile. This may have added to the completeness of our data. Overall, however, since we were able to use a large population with a long follow up and had significant results, this study is still able to shed light on the topic at hand. 

## 5. Conclusions

Our study suggested a strong association between early PTB and placental abruption. Since the most significant risk factor for placental abruption is a previous placental abruption, a future area for research may be looking at rates of early PTB due to abruption in those with previous placental abruption. Another interesting feature of placental abruption is that the risk factors for abruption differ in term versus preterm abruption, but the exact difference in mechanism is not well understood, leaving this area open to further investigation. 

## Figures and Tables

**Table 1:** Characteristics of general population.

Characteristic:	Extreme PTB: 24 + 0–27 + 6 Weeks	Very PTB: 28 + 1–31 + 6 Weeks	Moderate–Late PTB: 32 + 0- 36 + 6 Weeks	Term Birth: >37 + 0 weeks	*p*-Value *	*p*-Value **
*n*	1155	2490	25,344	304,732		
Maternal Age (mean ± SD)	28.07 ± 6.642	28.32 ± 6.362	28.40 ± 6.186	28.19 ± 5.798	0.176	0.499
Ethnicity: Jewish Bedouin	46.7	42.9	48.1	48.4	<0.001	0.268
	53.3	57.1	51.9	51.6		
Nulliparity	37.3	31.9	29.7	23.8	<0.001	<0.001
Previous PTB ***	27.5	30.1	28.8	11.1	<0.001	<0.001
Previous cesarean delivery	25.7	27.9	25.9	15.3	<0.001	<0.001
Recurrent pregnancy loss	8.7	7.8	6.6	4.6	<0.001	<0.001
Diabetes mellitus	2.3	6.1	7.9	5.1	<0.001	<0.001
Hypertension	9.1	14.8	12.2	4.4	<0.001	<0.001
In vitro fertilization	7.2	8.6	6.4	1.3	<0.001	<0.001
Ovulation induction	2.9	4.7	3.5	0.9		
Lack of prenatal care	13.5	12.4	8.4	9.0	<0.001	<0.001
Gestational diabetes mellitus	1.2	4.1	5.4	4.0	<0.001	<0.001
Preeclampsia	7	13.6	10.5	3.5	<0.001	<0.001
Placenta previa	3.6	3.3	2.1	0.2	<0.001	<0.001
Placental abruption	12.8	10.2	2.4	0.3	<0.001	<0.001
Cesarean delivery	16.1	19.1	18.2	11.7	<0.001	<0.001
Assisted delivery	0.2	0.5	1.6	3.3	<0.001	<0.001
Blood transfusion	7.8	6.5	3.6	1.3	<0.001	<0.001
Postpartum hemorrhage	1.0	0.5	0.4	0.6	<0.001	0.030
Multiple gestation	22.1	26.9	23.2	1.6	<0.001	<0.001
Neonate’s Gender:						
Male	53.0	53.1	52.4	50.8	<0.001	0.164
Female	47.0	46.9	47.6	49.2		
Small for gestational age neonate	14.2	6.2	4.3	4.7	<0.001	<0.001
5-min APGAR < 7	28.6	6.4	1.3	0.3	<0.001	<0.001
Umbilical Cord pH < 7	2.9	0.9	0.9	0.4	0.007	0.026
Intrapartum death	5.0	0.6	0.1	0.0	<0.001	<0.001

* *p*-value for multiple comparisons (all four groups); ** *p*-value for comparison between the extreme PTB and all other groups; *** This analysis was restricted to women with birth order >1. APGAR: The APGAR score (named after Dr. Virginia Apgar) is a universal scoring system use to assess newborns one minute and five minutes after they are born.

**Table 2:** Logistic regression results for extreme preterm group.

Characteristic	Adjusted Odds Ratio	95% Confidence Interval	Significance (*p*-Value)
Lack of prenatal care	2.019	1.694–2.407	<0.001
In vitro fertilization	1.338	1.038–1.723	0.024
Nulliparity	2.304	2.006–2.647	0.071
Previous cesarean delivery	1.444	1.212–1.720	<0.001
Diabetes mellitus	0.322	0.217–0.477	<0.001
Preeclampsia	1.033	0.816–1.307	<0.001
Placenta previa	5.884	4.149–8.344	<0.001
Recurrent pregnancy loss	1.815	1.468–2.245	<0.001
Placental abruption	20.606	17.006–24.969	<0.001
Induction of labor	1.410	1.220–1.626	<0.001
Multiple gestation	7.714	6.581–9.042	<0.001

**Table 3:** Risk factors in women with previous PTB.

Characteristic:	Extreme PTB: 24 + 0–27 + 6 Weeks	Very PTB: 28 + 1–31 + 6 Weeks	Moderate–Late PTB: 32 + 0–36 + 6 Weeks	Term Birth: >37 + 0 weeks	*p*-Value *	*p*-Value **
*n*	204	514	5173	26,046	–	
Ethnicity: Bedouin Jewish	64.7	62.8	58.1	63.5	<0.001	0.537
	35.3	37.6	41.9	36.5		
Previous cesarean delivery	31.9	40.3	36.9	29.0	<0.001	0.661
Diabetes mellitus	1.5	6.6	9.1	6.7	<0.001	0.002
Hypertension	12.7	17.9	12.3	5.3	<0.001	0.001
In vitro fertilization	4.4	4.7	3.7	1.3	<0.001	0.005
Ovulation induction	1.5	3.1	1.5	0.5		
Gestational diabetes mellitus	1.5	4.1	5.7	5.0	<0.001	0.018
Preeclampsia	11.3	15.4	9.5	3.6	<0.001	<0.001
Placenta previa	4.4	3.5	1.8	0.3	<0.001	<0.001
Placental abruption	14.2	11.5	2.8	0.4	<0.001	<0.001

* *p*-value for multiple comparisons (all four groups); ** *p*-value for comparison between the extreme PTB and all other group.

**Table 4:** Logistic regression results for women with previous PTB.

Characteristic	Adjusted Odds Ratio	95% Confidence Interval	Significance (*p*-Value)
Lack of prenatal care	2.174	1.391–3.399	0.001
In vitro fertilization	1.358	0.659–2.797	0.407
Previous cesarean delivery	1.013	0.746–1.378	0.932
Diabetes mellitus	0.162	0.051–0.510	0.002
Preeclampsia	1.888	1.193–2.991	0.007
Placenta previa	4.161	1.958–8.839	<0.001
Recurrent pregnancy loss	2.370	1.664–3.374	<0.001
Placental abruption	13.579	8.757–21.057	<0.001
Induction of labor	1.478	1.018–2.145	0.040
Multiple gestation	6.177	4.202–9.080	<0.001

**Table 5:** Risk factors for singleton pregnancies.

Characteristic:	Extreme PTB: 24 + 0–27 + 6 Weeks	Very PTB: 28 + 1–31 + 6 Weeks	Moderate–Late PTB: 32 + 0–36 + 6 Weeks	Term Birth: >37 + 0 Weeks	*p*-Value *	*p*-Value **
*n*	905	1829	19,508	299,814	-	
Ethnicity: Bedouin Jewish	53.3	58.0	53.9	51.7	<0.001	0.386
	46.7	42.0	46.1	48.3		
Nulliparity	35.4	29.1	28.9	23.8	<0.001	<0.001
Previous PTB	18.4	22.1	22.9	8.6	<0.001	
Previous cesarean delivery	17.6	19.5	19.5	11.7	<0.001	<0.001
Recurrent pregnancy loss	8.4	8.0	6.7	4.6	<0.001	<0.001
Diabetes mellitus	2.1	4.8	7.4	5.0	<0.001	<0.001
Hypertension	10.3	17.1	12.0	4.3	<0.001	<0.001
In vitro fertilization	2.6	2.3	2.1	1.1	<0.001	<0.001
Ovulation induction	1.0	1.4	1.1	0.8		
Lack of prenatal care	15.6	13.7	9.7	9.0	<0.001	<0.001
Gestational diabetes mellitus	1.0	3.2	4.9	4.0	<0.001	<0.001
Preeclampsia	8.2	15.8	10.2	3.4	<0.001	<0.001
Placenta Previa	4.1	4.1	2.5	0.2	<0.001	<0.001

* *p*-value for multiple comparisons (all four groups); ** *p*-value for comparison between the extreme PTB and all other groups.

**Table 6:** Logistic regression results for singleton gestation group.

Characteristic	Adjusted Odds Ratio	95% Confidence Interval	Significance
Lack of prenatal care	2.136	1.772–2.574	<0.001
In vitro fertilization	1.687	1.104–2.580	0.016
Nulliparity	2.226	1.902–2.605	<0.001
Previous cesarean delivery	1.592	1.314–1.929	<0.001
Diabetes mellitus	0.345	0.218–0.545	<0.001
Preeclampsia	1.432	1.117–1.837	0.005
Placenta previa	5.845	4.022–8.945	<0.001
Recurrent pregnancy loss	1.747	1.368–2.232	<0.001
Placental abruption	24.619	20.063–30.210	<0.001
Previous PTB	2.305	1.980–2.784	<0.001
Induction of labor	1.528	1.312–1.778	<0.001

**Table 7:** Risk factors for multiple gestations.

Characteristic:	Extreme PTB: 24 + 0–27 + 6 Weeks	Very PTB: 28 + 1–31 + 6 Weeks	Moderate–Late PTB: 32 + 0–36 + 6 Weeks	Term Birth: >37 + 0 Weeks	*p*-Value *	*p*-Value **
*n*	255	670	5888	4960	–	
Ethnicity: Bedouin Jewish	53.3	54.6	45.1	47.1	<0.001	0.031
	46.7	45.4	54.9	52.9		
Nulliparity	43.9	39.4	32.5	24.3	<0.001	<0.001
Previous PTB	14.9	16.6	12.3	7.1	<0.001	
Previous cesarean delivery	11.0	18.1	13.9	10.4	<0.001	0.436
Recurrent pregnancy loss	9.8	7.3	6.3	6.1	0.084	0.023
Diabetes mellitus	2.7	9.7	9.4	8.4	<0.001	<0.001
Hypertension	4.7	8.5	12.9	8.3	<0.001	0.002
In vitro fertilization	23.5	25.7	20.4	14.9	<0.001	0.106
Ovulation induction	9.4	13.9	11.6	8.9		
Lack of prenatal care	6.3	9.0	4.1	5.3	<0.001	0.308
Gestational diabetes mellitus	2.0	6.7	7.3	6.9	0.013	0.002
Preeclampsia	2.7	7.6	11.6	7.2	<0.001	<0.001
Placenta Previa	2.0	0.9	0.5	0.2	<0.001	<0.001
Placental Abruption	7.1	4.5	0.9	0.7	<0.001	<0.001

* *p*-value for multiple comparisons (all four groups); ** *p*-value for comparison between the extreme PTB and all other groups.

**Table 8:** Logistic regression results for multiple gestation group.

Characteristic	Adjusted Odds Ratio	95% Confidence Interval	Significance
Lack of prenatal care	1.396	0.823–2.368	0.216
In vitro fertilization	1.171	0.856–1.604	0.324
Nulliparity	2.483	1.855–3.323	<0.001
Previous cesarean delivery	0.909	0.589–1.401	0.664
Diabetes mellitus	0.280	0.131–0.598	0.001
Preeclampsia	0.229	0.107–0.490	<0.001
Placenta previa	4.775	1.779–12.819	0.002
Recurrent pregnancy loss	1.853	1.207–2.846	0.005
Placental abruption	7.467	4.398–12.677	<0.001
Previous PTB	2.113	1.435–3.112	<0.001
Induction of Labor	0.424	0.198–0.907	0.027

**Table 9:** Risk factors for Induced PTB.

Characteristic:	Extreme PTB: 24 + 0–27 + 6 Weeks	Very PTB: 28 + 1–31 + 6 Weeks	Moderate–Late PTB: 32 + 0- 36 + 6 Weeks	Term Birth: >37 + 0 Weeks	*p*-Value *	*p*-Value **
*n*	281	346	4153	70,615		
Ethnicity: Bedouin Jewish	44.8	55.2	42.7	38.5	<0.001	0.083
	55.2	44.8	57.3	61.5		
Nulliparity	40.6	32.7	42.0	39.4	<0.001	0.719
Previous PTB	13.5	16.2	17.4	6.2	<0.001	
Previous cesarean delivery	10.0	11.3	5.3	4.6	<0.001	<0.001
Recurrent pregnancy loss	7.8	7.5	6.6	4.8	<0.001	0.023
Diabetes mellitus	2.5	5.2	8.6	9.0	<0.001	<0.001
Hypertension	11.7	14.7	24.3	9.0	<0.001	0.313
In vitro fertilization	2.5	2.0	3.0	1.8	<0.001	0.672
Ovulation induction	1.8	2.0	2.3	1.4		
Lack of prenatal care	11.7	12.4	5.7	4.8	<0.001	<0.001
Gestational diabetes mellitus	1.4	3.2	5.8	6.9	<0.001	<0.001
Preeclampsia	8.9	13.9	21.3	7.5	<0.001	0.716
Placenta previa	1.4	0.3	0.2	0.1	<0.001	<0.001
Placental Abruption	7.1	7.2	1.8	0.3	<0.001	<0.001

* *p*-value for multiple comparisons (all four groups); ** *p*-value for comparison between the extreme PTB and all other groups.

**Table 10:** Logistic regression results for Induced PTB group.

Characteristic	Adjusted Odds Ratio	95% Confidence Interval	Significance
Lack of prenatal care	2.585	1.771–3.713	<0.001
In vitro fertilization	1.205	0.557–2.608	0.635
Nulliparity	1.331	1.024–1.729	0.032
Previous cesarean delivery	2.177	1.430–3.315	<0.001
Diabetes mellitus	0.262	0.123–0.556	<0.001
Preeclampsia	0.954	0.628–1.449	0.825
Placenta previa	9.193	2.867–29.484	<0.001
Recurrent pregnancy loss	1.611	1.030–2.521	0.037
Placental abruption	14.175	8.654–23.218	<0.001
History of PTD	2.013	1.383–2.292	<0.001

## Data Availability

The data presented in this study are available on request from the corresponding author. The data are not publicly available due to restrictions e.g., privacy or ethical.
